# Past speculations of the future: a review of the methods used for forecasting emerging health technologies

**DOI:** 10.1136/bmjopen-2015-010479

**Published:** 2016-03-10

**Authors:** Lucy Doos, Claire Packer, Derek Ward, Sue Simpson, Andrew Stevens

**Affiliations:** 1 NIHR Horizon Scanning Research and Intelligence Centre, Institute of Applied Health Research, University of Birmingham, Birmingham, UK; 2 Institute of Applied Health Research, University of Birmingham, Birmingham, UK

**Keywords:** Forecasting, health technology, innovations, methods, Systematic review

## Abstract

**Objectives:**

Forecasting can support rational decision-making around the introduction and use of emerging health technologies and prevent investment in technologies that have limited long-term potential. However, forecasting methods need to be credible. We performed a systematic search to identify the methods used in forecasting studies to predict future health technologies within a 3–20-year timeframe. Identification and retrospective assessment of such methods potentially offer a route to more reliable prediction.

**Design:**

Systematic search of the literature to identify studies reported on methods of forecasting in healthcare.

**Participants:**

People are not needed in this study.

**Data sources:**

The authors searched MEDLINE, EMBASE, PsychINFO and grey literature sources, and included articles published in English that reported their methods and a list of identified technologies.

**Main outcome measure:**

Studies reporting methods used to predict future health technologies within a 3–20-year timeframe with an identified list of individual healthcare technologies. Commercially sponsored reviews, long-term futurology studies (with over 20-year timeframes) and speculative editorials were excluded.

**Results:**

15 studies met our inclusion criteria. Our results showed that the majority of studies (13/15) consulted experts either alone or in combination with other methods such as literature searching. Only 2 studies used more complex forecasting tools such as scenario building.

**Conclusions:**

The methodological fundamentals of formal 3–20-year prediction are consistent but vary in details. Further research needs to be conducted to ascertain if the predictions made were accurate and whether accuracy varies by the methods used or by the types of technologies identified.

**Strengths and limitations of this study**

- Our study offers a comprehensive review of the methods used for forecasting in the healthcare field.
- Involvement of experts was the most common method used for forecasting emerging health technologies while complex methods such as scenario building and analysis were the least to be used.
- We excluded studies that were not in English.
- It remains unclear if the predictions made are precise and further scrutiny is needed to evaluate the accuracy of predictions made in terms of individual health technologies and timescale.

## Introduction

Forecasting comprises a set of tools used in strategy development. In health, it can be used to build strategies for future delivery of healthcare and to prepare health services for external pressures to change. In particular, it can support rational decision-making around the introduction and use of emerging health technologies and prevent investment in technologies that have limited long-term potential.^1–3^ Healthcare providers and payers need advance notice to allow for strategic planning of budgets, infrastructure requirements, staff training and recruitment.^4^

Japan led the widespread adoption of technology forecasting, conducting Delphi-style surveys on a 5-yearly basis from 1971 looking up to 30 years into the future for developments and innovations, including those in healthcare.^5^ In the UK, the Technology Foresight Programme^6^ aims to inform future thinking in government and includes topics relating to healthcare. Individual projects within the Foresight Programme have different aims, including setting out a vision for future research, assessing the role of future technologies in care and creating challenging visions of the future to ensure effective strategies are in place.^6^

Many forecasting methods have been described, some involving the use of experts, others adopting broader public participation.^7^ Methods used include expert panels, interviews and Delphi studies, trend analysis, driver analysis, scanning of literature and online sources, technology road mapping, and scenario building. Each method has its strengths and weaknesses and most forecasting programmes use a combination of approaches.^5^ ^8^

Forecasting methods need to be credible and the final forecasts need to be accurate, but accurate forecasting can be difficult and is likely to be influenced by how far ahead one is attempting to predict, the type of technologies being targeted, as well as the methods used. Although important, research into the accuracy of health technology forecasts and their impact on health service preparedness is scarce. Initial searches of published literature revealed only two studies that have evaluated the accuracy of forecasting for selected health technologies. Douw and Vondeling^9^ evaluated the ability of oncologists to assess the potential impact of emerging anticancer drugs over a 5-year period and concluded that experts were good at predicting which drugs were not expected to have a significant impact, but less good at predicting those that would have an impact, missing 37% of drugs that were in need of guidance.^9^ Lerner *et al*^4^ analysed four case studies to examine accuracy of predictions for four technologies: single-room proton beam radiation therapy for various cancers; digital breast tomosynthesis imaging technology for breast cancer screening; transcatheter aortic valve replacement for serious heart valve disease; and minimally invasive robot-assisted surgery for various cancers. They found that 5 out of 20 predictions concerning patient use, adoption status, health, finance and process impacts relating to the 4 health technologies in a prior forecasting exercise were inaccurate. The authors suggested that the inaccuracies arose from a lack of information on time to availability, atypical licensing and reimbursement decisions, and an inability to predict reimbursement rates. However, the authors did not investigate the consequences of inaccurate predictions.

As the first part of an evaluation of the accuracy of health technology forecasting, we undertook a narrative systematic review of the methods used to predict emerging health technologies within a 3–20-year timeframe. We selected this timeframe to distinguish from short-term predictions (less than 3 years), which are more likely to be in the form of horizon scanning exercises for technologies that are already in late-stage development when information is more certain. Conversely, we also anticipate that predictions made many decades ahead will not only tend to be inaccurate, but also likely to be of a quite different nature and not focused on specific individual or groups of novel health technologies.

## Methods

### Search strategy and data sources

We performed a systematic search to identify studies reporting methods used to predict future health technologies. We included studies that: (1) made predictions of named health technologies or groups of technologies; (2) reported an identified method (or methods) for making those predictions; (3) included some indication of timescale for the development, adoption or impact of the predicted technologies; and (4) had a prediction period of 3–20 years. We excluded commercially produced market research reports, long-term futurology studies (with minimum timeframes of more than 20 years), speculative editorials and opinion pieces that did not report a methodology for prediction, and studies looking at the near future (under 3 years).

We searched the bibliographical databases MEDLINE, EMBASE and PsycINFO, including articles published in English between 1947 and May 2014. Based on a scoping of the literature, our search terms included relevant MeSH headings and text words for (1) forecasting methods, (2) health technologies, (3) innovation and (4) study purpose, for instance forecasting, predicting or horizon scanning. The details of individual search strategies with the number of documents found are shown in online supplementary appendix S1. Regular updates were made to capture any new publications before manuscript submission.

10.1136/bmjopen-2015-010479.supp1 Supplementary appendix

Additional searches included checking the bibliography and reference lists of relevant articles, and hand searching of key journals published during the period 1980–2014 inclusive (*International Journal of Technology Assessment in Health Care, Health Affairs, Futures, International Journal of Forecasting, Foresight, Health Policy and the British Medical Journal*). We also searched the grey literature using relevant databases (OpenGrey^10^ and OAIster^11^), the Index to Theses,^12^ medical conference proceedings (ISI's Conference Proceedings Citation Index^13^ and Zetoc^14^), Google and Google scholar. We contacted collaborators from the EuroScan International Network^15^ to identify any unpublished reports, and attempted to contact report authors for missing information in order to apply our inclusion criteria.

### Data extraction

Two researchers (LD and CP) examined the titles and abstracts for relevance, and ordered all potentially relevant papers and reports. The two researchers independently examined the full documents and applied the inclusion and exclusion criteria. Disagreement about studies for inclusion was resolved by discussion between the two reviewers. For multiple reports by the same group using the same forecasting method, the report with the most recent and complete data was selected for inclusion. Repeated forecast exercises from these groups were counted as one study.

LD extracted data including study year, country, forecast method(s), forecast period and predicted health technologies for analysis. Given the nature of the study and the diversity of methods used to forecast, we present the results of studies in a narrative synthesis.

## Results

**Figure 1:**
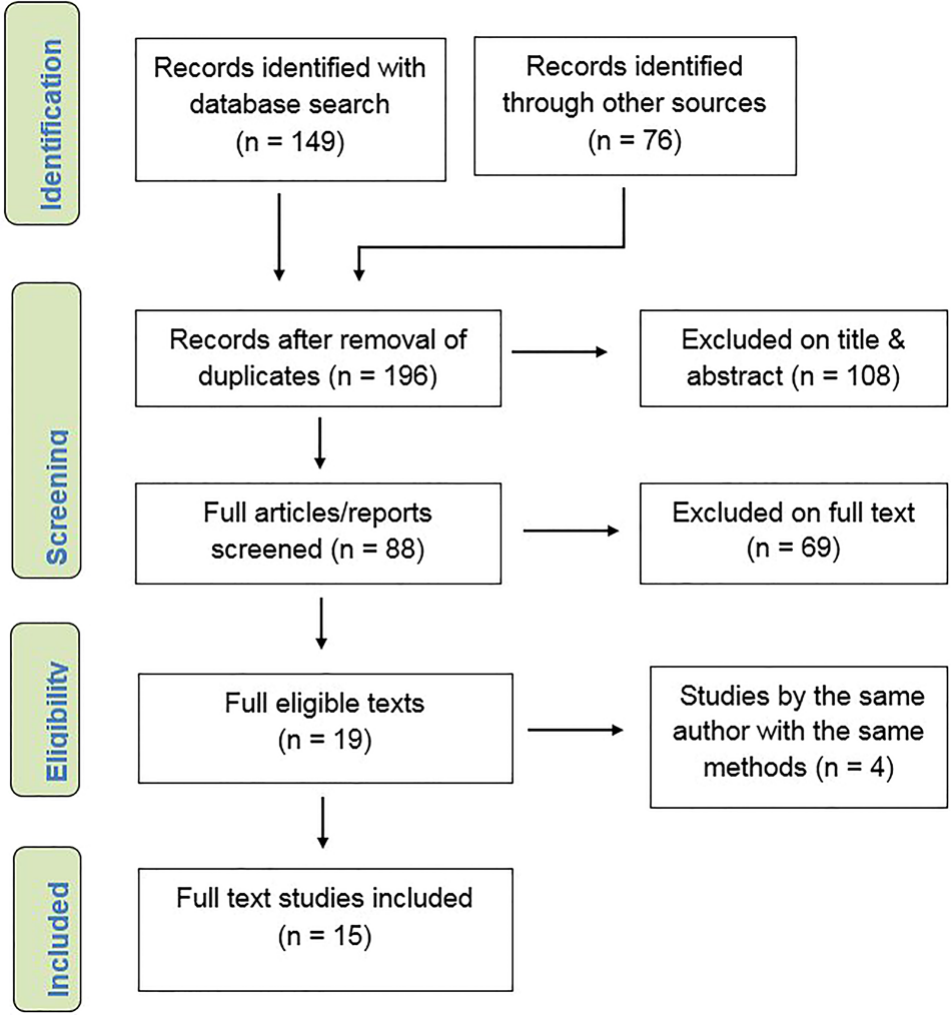
Flow chart of article/report selection process.

We initially identified 225 potentially relevant articles and reports, with 194 remaining after removal of duplicates. We discarded 108 abstracts that did not meet our inclusion criteria and rejected a further 69 articles after reading and reviewing the full text. After excluding 4 repeat studies by the same institution using the same forecast method(s), 15 studies met our inclusion criteria and were included in the analysis (figure 1).

### Characteristics of included studies

Table 1 describes the year and country of the study, period and method(s) of forecasting, organisation and the types of technologies targeted. Out of the 15 studies included in our review, 12 were from high-income countries (6 from the UK) and 2 were from middle-income countries (South Africa and China).^21^ ^25^ One international study used experts from around the world who were originally from developing countries, had worked in developing countries or were familiar with the public health problems of these countries.^24^ The majority of the included studies were performed at a national level, with only four studies including an international element.^16^ ^23^ ^24^ ^26^

**Table 1:** Characteristics of included studies with forecast period and methods used

Study name or first author	Forecast group, country	Year of forecast	Forecast period	Forecast method(s)	Type of experts and/or expertise	Types of technology identified
Dutch Steering Committee on Future Health Scenarios (STG)^16^ ^17^	Governmental organisation, The Netherlands	1986	4–15 years	Survey of experts in the Netherlands and other developed countries	Researchers, scientists and clinicians	All types of healthcare technologies
Spiby^18^	Researcher for a local hospital and health authority, UK	1988	Up to 20 years	Delphi study to experts	Expertise not specified	Health technologies in three main areas: diagnostic, therapeutic and information technology
Loveridge *et al*^19^	Policy research group for a governmental foresight programme, UK	1994	To 2015 (20 years)	Delphi survey to experts	No details of expertise available	Technologies in a variety of fields including health
Stevens *et al*^20^	Research group, UK	1995	No upper limit, but generally up to 5 years	Literature search Analysis of evidence from organisations with a role in health technology assessment coordination Postal survey of experts	Clinicians, public health experts and others	All types of healthcare technologies
Karim, South Africa's National Research and Technology Foresight Project^21^	Governmental organisation, South Africa	1996	10–20 years	Survey of experts Expert workshops Scenario building and analysis Identifying national and international studies	No details of expertise available	Technologies in 12 sectors including health
Rosin and Kemp: operating theatre of the year 2010; Department of Trade and Industry report^22^	Governmental organisation, UK	1996	15 years	Working group of experts	From industry and medicine	Technologies for use in the operating theatre
Cahill and Scapolo^23^	Research group for the European Commission Institute for Prospective Technological Studies, Europe	1998	To 2010 (12 years)	Review of national technology foresight studies		Technologies in 6 areas including life sciences
Daar *et al*^24^	Research group, supported by health research institutes and a WHO Collaborating Center, developing countries	2002	5–10 years	Delphi survey to scientific experts	Scientists and public health experts	Biotechnologies for improving health in developing countries
Technology foresight towards 2020_China^25^	Research group in the Chinese Academy of Sciences, China	2003	To 2020 (17 years)	Scenario building for identifying technology demands Delphi survey to experts	Technology and administrative experts	Technologies in 4 fields including biotechnology and medicine
British Telecommunications (BT) calendar (BT calendar 2005: A report by the British Telecommunication. Personal communication with the editor Ian Pearson 2014)	Commercial organisation, UK	1997 and 2005	Short term (1–5 years) to very long (up to 2045, 48 years) for 1997 survey; up to 2050 (45 years) for 2005 survey	Searches of literature and the internet The authors used their own judgement where they could not find articles in journals Chats with world experts	Engineers and others	Ranged from technologies related to money and finance to computers and IT. Included biotechnology, health and medical technologies
Tremblay and Yiu^26^	Consultancy agency contracted by regional health organisations, Canada	2006	To 2020 (15 years)	Literature review of foresight studies and technology horizon scans conducted by governments and national science and technology centres		Diagnostic technologies
Food and Drug Administration (FDA) surveys^27^	Governmental organisation, USA	1998 and 2008	Up to 10 years	Delphi surveys to FDA and non-FDA experts Interviews with experts Expert workshop	Clinicians, engineers, managers and others	Medical device technologies
Institute of the Future, 2009^28^	Independent non-profit research organisation, USA	2009	To 2020 (11 years)	Survey of experts from the institution and outside	Scientists, academics and clinicians	Technologies in health and healthcare
Science and Technology Foresight Survey Japan, 2010^29^	Governmental organisation, Japan	Every 5 years from 1971, latest survey 2009–2010	From 5–10 years to 30 years	Delphi survey to experts	Researchers, industry and others	Technologies in a variety of fields including health
UK technology and innovation futures for the 2020s, 2010^30^	Governmental organisation, UK	2010	In the 2020s (10–20 years)	Survey of experts Expert workshops Interviews with experts	Researchers, industry and others	Technologies from clusters including health and medicine

Seven of the studies were undertaken by governmental agencies, six by researchers and research groups, and two by other organisations. Eight of the studies were forecasts of healthcare technologies only, whereas the remaining seven studies considered a variety of technologies, including healthcare.

### Forecast period

The forecast period varied from short to medium time periods of 3–10 years, to long-term forecasts into future decades. However, the studies with the longest forecasts also included forecasts for shorter timescales that met our inclusion criteria.

Three of the studies in our review have been repeated either once or regularly. The Technology Foresight survey in Japan^29^ has been repeated every 5 years from 1971. The Food and Drug Administration (FDA) survey^27^ in the USA was carried out in 1998 and 2008, and the British Telecommunication (BT) calendar forecast^26^ in the UK was undertaken for reports in 1997 and 2005. The UK technology innovation futures 2020^30^ was initially carried out in 2010 and then refreshed (rather than repeated) in 2012. Other studies report one-off exercises and have not been repeated as far as we are aware.

### Methods of forecasting

Thirteen studies in our review used similar processes to identify future developments in health technologies; that is consulting experts, either alone or in combination with other methods, such as literature searching and scenario development and analysis. Overall, surveys of experts (including Delphi studies) was the most common method of forecasting, used in 10 studies (in half of which it was the only method used, table 2). Based on the information provided on methods, all of the studies that exclusively set out to identify health technologies used clinicians within the groups of experts consulted. We had insufficient information to ascertain whether the more general forecast studies that used experts had included clinicians or not. Reviews of the literature for biomedical studies and other published reports were the second most common method, being used in five studies (in 2 of which it was the only method used).

**Table 2:** Number of studies using each method either alone or in combination

Forecasting method	Single (n=9)	Combined (n=6)	Total (n=15)
Expertise (n=16)			
Authors as experts	0	1	1
Interviews	0	3	3
Surveys	2	3	5
Delphi	3	2	5
Workshops	0	3	3
Expert panel	2	0	2
Evidence based (n=6)			
Review of literature	2	3	5
Evidence from HTA organisations	0	1	1
Creativity based (n=2)			
Scenario analysis and building	0	2	2

HTA, Health Technology Assessment.

**Figure 2:**
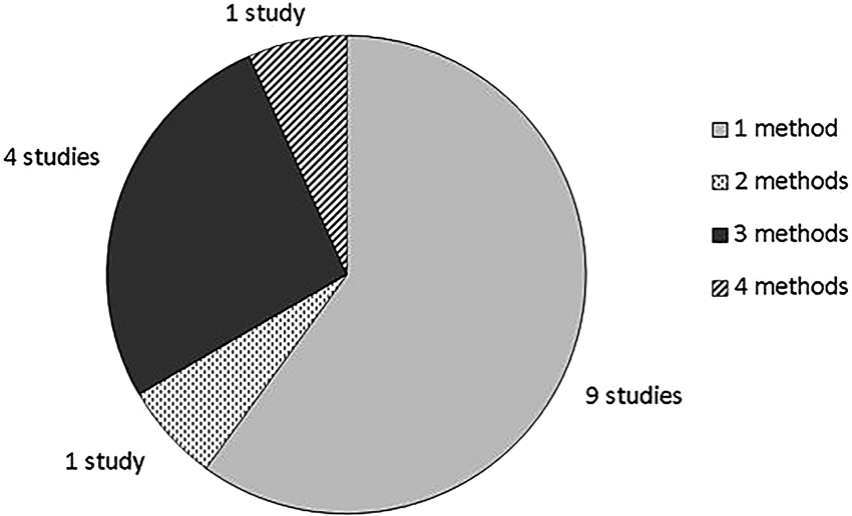
Number of forecasting methods used.

The majority of the studies (9/15) used a single method of forecasting (figure 2), 7 of which involved consulting experts. Among studies using a single method of forecasting, the Delphi technique was the most common method used (3/9), followed equally by surveys of experts (2/9) and review of the literature and published reports (2/9). All of the studies that used more than one method of forecasting included consulting experts as one of their methods. The Technology Foresight towards 2020 in China^25^ used two methods: scenario building and Delphi surveys. Four studies (4/15) used three methods of forecasting (BT calendar 2005: A report by the British Telecommunication. Personal communication with the editor Ian Pearson 2014).^20^ ^27^ ^30^ The study by Karim^21^ was the only study that used four methods: a review of the literature, a survey of experts, expert workshops and scenario analysis.

## Discussion

### Main findings

Our review found that the methods reportedly used to forecast health technologies and trends in healthcare that will impact on strategic planning, decision-making and investment in healthcare systems in the medium-term to long-term were very similar. We found that methods incorporating the involvement of experts were the most common approach used by the identified studies, followed by reviewing the literature. Experts were engaged in different ways, with surveys and Delphi studies being the most commonly used methods. We found only two studies that used more complex methods, scenario analysis and scenario building (both alongside the use of experts), and no studies that used publications on patent applications, trend analysis, driver analysis or technology road mapping.

The extensive use of surveys and Delphi studies to engage experts is not unexpected, given the increased costs and organisational complexity required to arrange interviews, workshops and panels. Both methods are relatively cheap and quick to conduct, and can engage a large number of potentially diverse experts. In addition, the Delphi technique enables moderation of expert views and the development of new questions and areas of interest during the feedback rounds.^18^ In contrast, more complex methods require specialist skills to administer and can consequently be costly. These techniques may therefore be more appropriate for well-funded, comprehensive forecasting projects.

### Interpretation in the light of other literature

Eight of the studies used expert engagement as a single method. This single-source approach is not supported by commentators in the literature who recommend that forecasting projects employ more than one method.^8^ ^31^ ^32^ Sun and Schoelles^33^ reporting discussions from a panel of experts in horizon scanning (rather than forecasting), noted that “experts’ opinions should not substitute for comprehensive, proactive searches of other sources of information” when assessing the potential impact of health technologies.

Research suggests that using a multiround process, such as the consensus method used in Delphi studies, can improve the accuracy of forecasts, as does the experts’ level of expertise.^8^ ^34^ Tichy^34^ analysed the German 1993 and Austrian 1998 technology Delphi studies and found that experts who rated themselves as most knowledgeable tended to be more optimistic than those experts who rated themselves as somewhat less knowledgeable. Other factors identified by authors as relevant to the success of using surveys to consult experts include selecting the right mix of experts with different levels and breadth of experiences, asking them appropriate questions (eg, questions of clear importance and relevance), and an effective analysis of the results to deepen the understanding of emerging themes.^7^ ^34^ We did not have sufficient information to explore the type and level of expertise used within our included studies and only one study stated that they omitted the responses from experts who stated that they were unfamiliar with a specific technology or group of technologies under deliberation.^24^ The impact of potential conflicts of interest may also need consideration.^34^

Our finding, that reviewing the literature was also a commonly used forecasting method, is in keeping with the importance given to this approach by others undertaking forecasting in non-health fields. The European Agency for Safety and Health at Work^8^ concluded that literature reviews are crucial to establish the knowledge base for any forecasting project and to help identify gaps in knowledge. However, such reviews can be lengthy and the time taken for publication can undermine the contemporary value of the forecasts.^8^

Given the complexity and diversity of approaches to developing and applying scenarios, it was not surprising that we found only two studies that used scenario analysis and building, and that in both cases it was combined with other methods. This combined approach is supported by commentators, who suggest that the effectiveness and utility of scenario analysis can be enhanced by combining it with other methods such as the Delphi technique.^35^

### Strengths and limitations of the study

Our study offers a unique and valuable review of the methods used for forecasting in the healthcare field, and the data set can form the basis for additional work on the accuracy of individual forecasting methods. However, although we used a wide range of search strategies to identify studies, we found only a small number of forecasts in the healthcare field with replicable methods that met our timeframe criteria and that listed individual or groups of health technologies.

A limitation of our search strategy was the exclusion of studies published in languages other than English. We also surmise that many forecast projects, particularly the more regional or local studies, may not be published in a formal sense, remaining as internal reports that we could not identify or access. Similarly, it may also be that earlier projects, such as those undertaken prior to the widespread adoption of the internet, may be less likely to have been published in an accessible format. We have no reason to believe, however, that such studies would have used a different range of forecasting methods to the studies we found. Finally, by restricting our review to a 3–20-year timeframe, it could be argued that we will have missed forecasting projects with very long time frames. We believe, however, that such long-term speculative studies would have had quite different aims to those we identified, with a focus away from specific individual health technologies.

### Unanswered questions and further research

Given the potential cost of broad-ranging forecasting programmes, it is important to consider the accuracy of the forecasts as well as the impact of forecasts on the support given to the development of innovations; the development of relevant research, business support and health policies; and/or the commissioning of health services. We have not yet considered the accuracy of predictions made in the identified studies. In view of the extensive use of experts in these studies, further analysis will need to incorporate considerations of the selection processes for experts, the questions used and the methods employed to combine opinions. In addition, when evaluating the impact of forecasting exercises, effects on business, research or health system readiness to react to or adopt innovations may prove more important than precisely measuring the accuracy of predictions made about individual health technologies.

## Conclusions

Over the past few decades, technology forecasting has been promoted as a way of informing the strategic decision-making process in many areas of national, regional and local government. However, accurate forecasting is difficult and potentially costly; consequently identifying the most accurate and efficient methods of forecasting for different time horizons and technology fields is important. This study finds that consulting experts is the main, and often the sole, method of forecasting in the field of health technologies, with more complex forecasting methods being rarely used for this purpose. Plans for future research include consideration of how the accuracy (or impact) of predictions can be judged, and using our data set to evaluate the accuracy of predictions made in terms of individual health technologies, timescales and the methods used.
