# Supplementary material for: Past speculations of the future: a review of the methods used for forecasting emerging health technologies
Source: BMJ Open. 2016 Mar 10;6(3):e010479. doi: 10.1136/bmjopen-2015-010479 (PMC4800127; doi:10.1136/bmjopen-2015-010479)
Supplement: Supplementary appendix [file bmjopen-2015-010479supp_appendix.pdf]

Appendix 1: Search strategy

Ovid MEDLINE: 1946 to April Week 4 2014

Search no.	Keyword(s)	Number of hits
1	*Forecasting/mt [Methods]	497
2	*focus groups/ or exp questionnaires/	305537
3	1 or 2	306014
4	*biomedical technology/ or exp biomedical enhancement/ or nanomedicine/ or exp biotechnology/ or exp inventions/ or man-machine systems/ or exp miniaturization/ or technology, dental/ or exp technology, pharmaceutical/ or technology, radiologic/	212461
5	exp Diagnosis/td, ut [Trends, Utilization]	34815
6	exp therapeutics/ or exp "anesthesia and analgesia"/ or exp surgical procedures, operative/ or exp investigative techniques/ or exp dentistry/ or exp "equipment and supplies"/	13020454
7	4 or 5 or 6	13041556
8	Innovation.mp.	43864
9	(predict* or speculat* or foretell*).mp. [mp=title, abstract, original title, name of substance word, subject heading word, keyword heading word, protocol supplementary concept word, rare disease supplementary concept word, unique identifier]	930724
10	horizon scan*.mp. [mp=title, abstract, original title, name of substance word, subject heading word, keyword heading word, protocol supplementary concept word, rare disease supplementary concept word, unique identifier]	68
11	9 or 10	930782
12	3 and 7 and 8 and 11	127

Embase Classic+Embase 1947 to 2014 Week 18

Search no.	Keyword(s)	Number of hits
1	*Forecasting/mt [Methods]	0
2	*focus groups/ or exp questionnaires/	426930
3	1 or 2	426930
4	*biomedical technology/ or exp biomedical enhancement/ or nanomedicine/ or exp biotechnology/ or exp inventions/ or man-machine systems/ or exp miniaturization/ or technology, dental/ or exp technology, pharmaceutical/ or technology, radiologic/	1289151
5	exp Diagnosis/td, ut [Trends, Utilization]	0
6	exp therapeutics/ or exp "anesthesia and analgesia"/ or exp surgical procedures, operative/ or exp investigative techniques/ or exp dentistry/ or exp "equipment and supplies"/	12992766
7	4 or 5 or 6	13365037
8	Innovation.mp.	20849
9	(predict* or speculat* or foretell*).mp. [mp=title, abstract, original title, name of substance word, subject heading word, keyword heading word, protocol supplementary concept word, rare disease supplementary concept word, unique identifier]	1264600
10	horizon scan*.mp. [mp=title, abstract, original title, name of substance word, subject heading word, keyword heading word, protocol supplementary concept word, rare disease supplementary concept word, unique identifier]	131
11	9 or 10	1264704
12	3 and 7 and 8 and 11	22

PsycINFO 1967 to April Week 4 2014

Search no.	Keyword(s)	Number of hits
1	*Forecasting/mt [Methods]	0
2	*focus groups/ or exp questionnaires/	14302
3	1 or 2	14302
4	*biomedical technology/ or exp biomedical enhancement/ or nanomedicine/ or exp biotechnology/ or exp inventions/ or man-machine systems/ or exp miniaturization/ or technology, dental/ or exp technology, pharmaceutical/ or technology, radiologic/	5215
5	exp Diagnosis/td, ut [Trends, Utilization]	0
6	exp therapeutics/ or exp "anesthesia and analgesia"/ or exp surgical procedures, operative/ or exp investigative techniques/ or exp dentistry/ or exp "equipment and supplies"/	334
7	4 or 5 or 6	5549
8	Innovation.mp.	15042
9	(predict* or speculat* or foretell*).mp. [mp=title, abstract, original title, name of substance word, subject heading word, keyword heading word, protocol supplementary concept word, rare disease supplementary concept word, unique identifier]	330235
10	horizon scan*.mp. [mp=title, abstract, original title, name of substance word, subject heading word, keyword heading word, protocol supplementary concept word, rare disease supplementary concept word, unique identifier]	9
11	9 or 10	330244
12	3 and 7 and 8 and 11	0
